# Carminic Acid Stabilized with Aluminum-Magnesium Hydroxycarbonate as New Colorant Reducing Flammability of Polymer Composites

**DOI:** 10.3390/molecules24030560

**Published:** 2019-02-03

**Authors:** Anna Marzec, Bolesław Szadkowski, Jacek Rogowski, Waldemar Maniukiewicz, Dariusz Moszyński, Przemysław Rybiński, Marian Zaborski

**Affiliations:** 1 Institute of Polymer and Dye Technology, Faculty of Chemistry, Lodz University of Technology, Stefanowskiego 12/16, 90-924 Lodz, Poland; boleslaw.szadkowski@edu.p.lodz.pl (B.S.); marian.zaborski@p.lodz.pl (M.Z.); 2 Institute of General and Ecological Chemistry, Lodz University of Technology, Zeromskiego 116, 90-924 Lodz, Poland; jacek.rogowski@p.lodz.pl (J.R.); waldemar.maniukiewicz@p.lodz.pl (W.M.); 3 Faculty of Chemical Technology and Engineering, West Pomeranian University of Technology, Szczecin, Piastów Ave. 42, 71-065 Szczecin, Poland; dmoszynski@zut.edu.pl; 4 Department of Management and Environmental Protection, Jan Kochanowski University, Zeromskiego 5, 25-369 Kielce, Poland; przemyslaw.rybinski@ujk.edu.pl

**Keywords:** carminic acid, hybrid materials, aluminum-magnesium hydroxycarbonate, flame retardancy, ethylene-norbornene copolymer

## Abstract

In this study, hybrid pigments based on carminic acid (CA) were synthesized and applied in polymer materials. Modification of aluminum-magnesium hydroxycarbonate (LH) with CA transformed the soluble chromophore into an organic-inorganic hybrid colorant. Secondary ion mass spectroscopy (TOF-SIMS), X-ray photoelectron spectroscopy (XPS), X-ray diffraction (XRD), thermogravimetric analysis (TGA), scanning electron microscopy (SEM), and UV-Vis spectroscopy were used to study the structure, composition, and morphology of the insoluble LH/CA colorant. Successful modification of the LH was confirmed by the presence of interactions between the LH matrix and molecules of CA. XPS analysis corroborated the presence of CA complexes with Mg^2+^ ions in the LH host. The batochromic shift in UV-Vis spectra of the organic-inorganic hybrid colorant was attributed to metal-dye interactions in the organic-inorganic hybrid colorants. Strong metal-dye interactions may also be responsible for the improved solvent resistance and chromostability of the modified LH. In comparison to uncolored ethylene-norbornene copolymer (EN), a modified EN sample containing LH/CA pigment showed lower heat release rate (HRR) and reduced total heat release (THR), providing the material with enhanced flame retardancy.

## 1. Introduction

Natural and synthetic dyes are used in a wide range of everyday products [1,2]. Their high solubility, as well as low thermal, chemical, and photo stability, strictly limit their use to certain materials and applications. Recently, interest has grown in new organic-inorganic colorants based on natural dyes [3,4,5,6,7,8,9]. This has led to the search for new pigments, formed by the immobilization of dyes in resistant substrates such clays, silicas, or zeolites [10,11,12,13,14]. Conventionally, hybrid pigments are obtained by the complexation of dye molecules with the metallic cations present in inorganic substrates, such as alumina or calcium carbonate. These forms of organic-inorganic pigments are called lakes [15,16]. By combining natural or synthetic dyes with inorganic structures, it is possible to obtain hybrid pigments with enhanced chemical properties and a better thermal stability.

One of the oldest known coloring agents is carminic acid (CA), which is obtained from the female *Coccus laccae* (*Lacciferlacca* Kerr) insect [17,18]. In its pure form, CA is extremely soluble in water and has a poor photo and thermal stability. Numerous studies have investigated ways to stabilize organic-inorganic pigments based on this chromophore. Pérez et al. [19] developed a new family of hybrid pigments by the combination of γ-Al_2_O_3_ and organic chromophores, such as CA, alizarin, purpurin, curcumin, and betacyanins. Spectroscopic analysis proved the formation of aluminum complexes between the aluminum mineral and particular organic groups (carboxylic acid, quaternary ammonium, and β-keto enol) present in the chromophores. Fournier et al. [20] reported the stabilization of carminic acid using montmorillonite. They emphasized the role of ketone and catechol functions during the formation of the lake pigment. Guillermin et. al. [21] found that the adsorption complex of CA with Al-pillared montmorillonite enhanced the photostability of CA molecules. Velho et. al. [22] described the encapsulation of a series of natural dyes, including CA, in a silica matrix via the sol-gel process with the use of alkoxides. Colorimetric analysis revealed that silica-encapsulated natural dyes exhibited a higher resistance to discoloration under weathering conditions in comparison to non-capsulated colorants. 

Recently, several studies have been devoted to the adsorption or intercalation of synthetic dyes based on the azo or anthraquinone chromophore into or onto layered double hydroxides [23,24,25,26,27]. This approach may lead to an enhanced resistance of organic dyes to solar irradiation, as well as an increased resistance to oxidative and thermal decomposition. However, there has still been little research into the stabilization of natural dyes using aluminum-magnesium hydroxycarbonate (LH).

In this work, we produced organic-inorganic pigment by the precipitation of CA onto LH. The new hybrid colorants were studied using a range of experimental techniques, including XRD, TOF-SIMS, X-ray photoelectron spectroscopy, thermal analysis, SEM, and UV-Vis spectroscopy. The chromostability of the new LH pigments was tested in terms of their resistance to thermal treatment and towards different solvents. The hybrid pigment was added as a colorant to ethylene-norbornene copolymer and the flammability of the resulting composite was investigated using the cone calorimeter test.

## 2. Results

### 2.1. X-Ray Photoelectron Spectroscopy (XPS)

**Figure 1:**
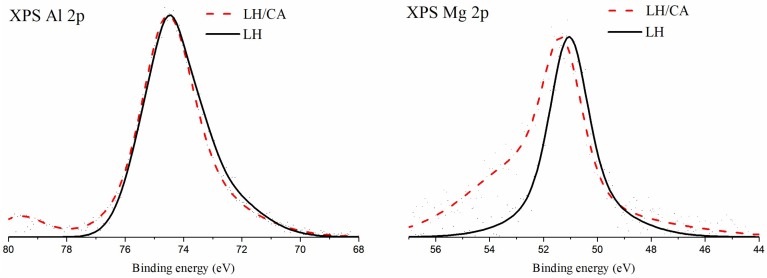
X-ray photoelectron spectra of Al 2p transition (**left** panel) and Mg 2p transition (**right** panel) acquired for aluminum-magnesium hydroxycarbonate (LH) before (black line) and after (red line) modification with carminic acid (20%).

**Figure 2:**
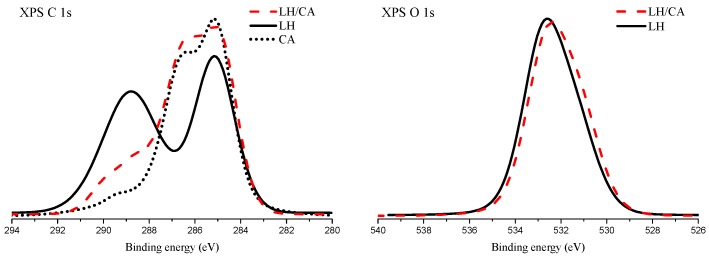
X-ray photoelectron spectra of C 1s transition (**left** panel) and O 1s transition (**right** panel) acquired for aluminum-magnesium hydroxycarbonate (LH) before (black line) and after (red line) modification with carminic acid (20%). XPS C 1s spectrum of pure carminic acid is depicted by dotted plot.

X-ray photoelectron spectroscopy was used to analyze the chemical state of the surface of the pure LH material, as well as of the hybrid with CA (Figure 1 and Figure 2, Table 1). The formation of chemical bonds between CA and magnesium or aluminum ions present in the LH structure should be reflected as a shift of the core-level electron states of these elements. The X-ray photoelectron spectra of the binding energy regions corresponding to Mg 2p and Al 2p are shown in Figure 1.

For both the pure LH and the hybrid material, the maximum of the XPS Al 2p peak is located at a binding energy of 74.4 eV. A binding energy close to 74 eV for the Al 2p line is attributed to a series of aluminum compounds consisting of Al-O bonds [28]. The chemical shifts for these compounds were minor. However, the Al 2p line positions for the Al_2_O_3_ type of bond are usually reported to be above 74 eV. The asymmetry observed in this line on the low-binding energy side could indicate the presence of an Al-O binding component with some contribution of hydroxyl groups. No notable difference was observed between the XPS Al 2p spectra acquired for pure LH and those for the hybrid with CA. Therefore, it may be concluded that there was no significant bonding between the aluminum ions in LH and the CA molecules.

The XPS Mg 2p peak observed for pure LH is symmetrical and its maximum is located at a binding energy of 50.9 eV. The position of the Mg 2p line characteristic for Mg-O biding in MgO oxide has been reported as being in the binding energy range of between 50.4 eV and 50.8 eV [29,30]. The respective positions of this line corresponding to MgCO_3_ and Mg(OH)_2_ bonds are given as being in the range of 51.0 eV to 51.4 eV [31]. Aluminum-magnesium hydroxycarbonate (LH) contains magnesium ions coordinated with oxygen ions, as well as carbonate and hydroxyl ions. Each of these chemical environments can be presumed to have contributed to the envelope of the XPS Mg 2p peak. 

After modification with CA, the maximum of the Mg 2p line shifted to 51.4 eV. A similar shift of the Mg 2p line has also been reported in the case of a hybrid of LH with alizarin [32]. This shift was assumed to have resulted from the formation of bonds between dye molecules and magnesium ions. A similar mechanism may occur in the present system. The prominent asymmetry on the high-energy side of the Mg 2p line extends to the binding energy region, which is above the typical range ascribed to Mg 2p transition. It is possible that some differential charging of the LH particles takes place.

Figure 2 presents the XPS spectra of the C 1s and O 1s transitions for the considered samples. Two distinctive peaks are present in the XPS C 1s spectrum of aluminum-magnesium hydroxycarbonate (LH). The maximum of the first peak is located at 285.1 eV and is ascribed to “adventitious carbon” present on the surface of the sample. The second peak is very broad and relatively intense, with the center of gravity placed at 289.0 eV. This peak marks the presence of carbonate ions from the aluminum-magnesium hydroxycarbonate (LH) structure. After the modification of aluminum-magnesium hydroxycarbonate with carminic acid, the XPS C 1s spectrum is changed considerably. Three different local maxima can be distinguished. The most intense one is located at 285.0 eV and is attributed to C-C and C-H bonds. The origin of this transition corresponds to the presence of the aliphatic bond present in carminic acid molecules. The second local maximum is located at 286.5 eV and can be attributed to the presence of C-OH bonds. Several C-OH bonds are present in the carminic acid molecule. Therefore, this spectrum feature is considered as an indicator of CA molecules. The XPS C 1s spectrum of pure carminic acid is shown for comparison in the left panel of Figure 2. The parts of the XPS C 1s spectra of the pure CA and LH/CA hybrid shown in the binding energy range between 282 eV and 287 eV are very similar. This observation can be taken as proof of the presence of carminic acid on the surface of the LH/CA hybrid sample. At the binding energy of 289 eV, a shoulder is observed in the XPS C 1s spectrum of the LH/CA hybrid. Presumably, this is a superposition of the XPS signal from the carbonate ions in the LH structure and the carboxyl functional groups from carminic acid. 

Figure 2 also shows the XPS O 1s spectrum for a pure LH sample as well as for the LH/CA hybrid. The envelopes of these spectra are very similar, with maxima at about 532.5 eV. This binding energy region is typical for the presence of Al-O and C-OH bonds. Some asymmetry in the O 1s peaks in the region of low binding energy can be ascribed to the presence of Mg-O bonds.

A substantial increase was observed in the concentration of carbon atoms on the surface of the LH/CA sample, in comparison to the LH sample. The ratios between the concentrations of aluminum and magnesium atoms were virtually identical. This effect may be explained by the screening of Al and Mg atoms from the LH structure by the CA molecules adsorbed on top of the LH structure. 

### 2.2. Secondary Ion Mass Spectrometry (TOF-SIMS) 

**Figure 3:**
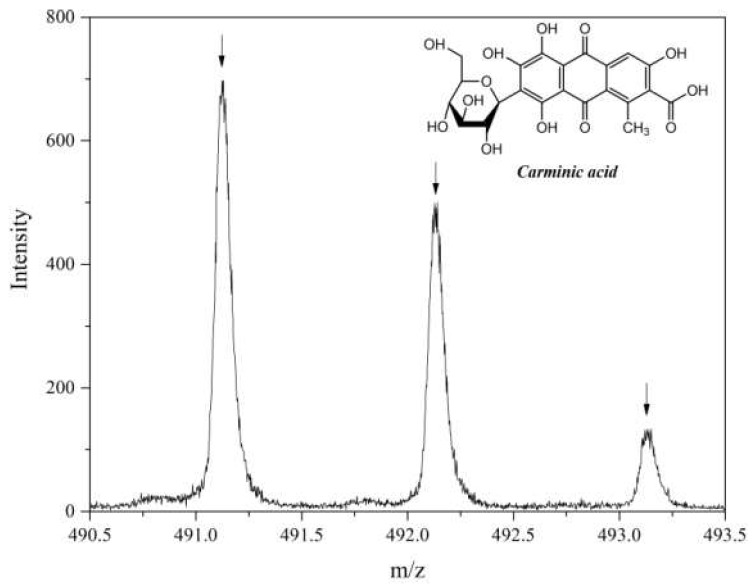
TOF-SIMS spectra of (C_22_H_20_O_13_-H)^−^ ion emitted from carminic acid.

Figure 3 shows the TOF-SIMS spectrum of negative ions for pure CA. The pseudomolecular ion (C_22_H_20_O_13_-H)^−^ and the fragmentation ion C_16_H_9_O_8_^−^ formed the most intense peaks in this spectrum. However, none of these ions appeared in the TOF-SIMS spectrum of the LH modified with CA. This may indicate that the interaction between the CA molecules and the host matrix was so strong that they could not be desorbed from the sample during TOF-SIMS spectrum acquisition. 

### 2.3. X-Ray Diffraction Analysis (XRD)

**Figure 4:**
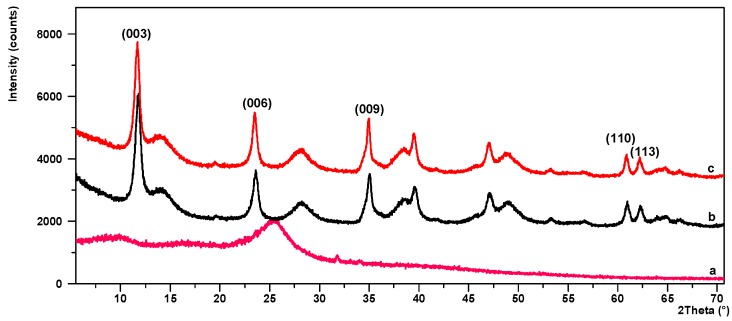
Powder diffraction patterns for CA (**a**), LH (**b**), and LH/CA (20%) (**c**).

Figure 4a–c shows XRD patterns for CA, LH, and LH/CA. As can be seen from the diffraction pattern in Figure 4a, CA is an amorphous substance. In contrast, the XRD pattern of the LH sample (Figure 4b) shows characteristic sharp and symmetrical peaks at low 2*θ* values, which are ascribed to diffractions by planes (003) and (006). These correspond to basal spacing and higher order diffractions [33]. The interlayer distances of (d_003_) and (d_006_) are 0.751 nm and 0.377 nm, respectively. These distances are characteristic for crystalline hydrotalcite-like compounds, which exhibit hexagonal lattices. On the slopes of the sharp peaks, small broad peaks can be observed, which may have been caused by unrecognized crystalline impurities. After the addition of CA, the basal reflection (003) of the LH/CA pigment (Figure 4c) shifted slightly to a lower 2*θ* angle, but this did not significantly affect the distance between the layers.

### 2.4. Thermogravimetric Analysis (TGA)

**Figure 5:**
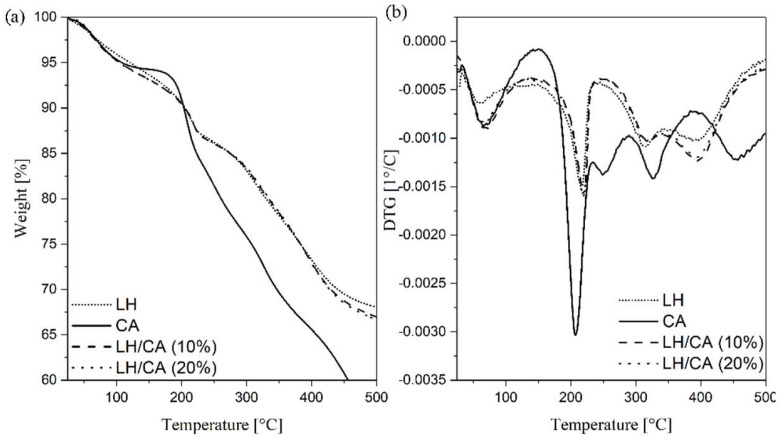
TGA (**a**) and DTG (**b**) profiles of CA, LH, LH/CA (10%), and LH/CA (20%).

The TG-DTA curves for pure CA, LH host, and the hybrid pigment were analyzed to investigate their thermal behavior. The thermal decomposition of LH/CA (10%) and LH/CA (20%) (Figure 5a,b) can be seen to have proceeded in four weight loss steps. The first, in a range from room temperature to 100 °C, corresponds to the loss of physically adsorbed water. The second weight loss (100–215 °C) is related to the removal of intercalated water molecules (dehydration) in the interlayered galleries. The relatively sharp third transition at 215–315 °C is due to removal of hydroxyl groups (dehydroxylation) from the LH layers as water molecules. The small peak at approximately 334 °C can be attributed to the partial loss of OH^−^ in the brucite-like layer. The final step (350–450 °C) is mostly associated with the liberation of carbonate ions inside the LH galleries and the beginning of hydroxy layer decomposition [34]. This step is often characterized by the complete decomposition of metal hydroxide layers and the formation of metal oxides. In the case of CA, the first mass loss is caused by the loss of water, whereas the maximum mass loss rate of the carminic chromophore occurred at 200 °C [35]. The *T_5%_* values of the LH-based pigments were found to be lower (105 °C) in comparison to the CA chromophore (107 °C) and pure LH host (119 °C) (Table 2). It is interesting to note that the 20% weight loss temperatures for LH/CA (10%) (334 °C) and LH/CA (20%) (335 °C) were slightly shifted to a higher temperature than in the case of pure LH (329 °C), probably as a result of CA incorporation. However, the decomposition of carminic acid is not observed on the thermograms for the hybrid pigment. This behavior can be explained by the fact that the main weight loss peaks for the CA dye were similar to those for dehydroxylation and carbonate decomposition in the LH host. These results also correspond with the observations made in our previous works [36], in which the decomposition peaks of organic chromophores incorporated in an LH matrix were covered by peaks signaling inorganic carrier decomposition.

### 2.5. Color Stability of LH/CA Pigments

**Figure 6:**
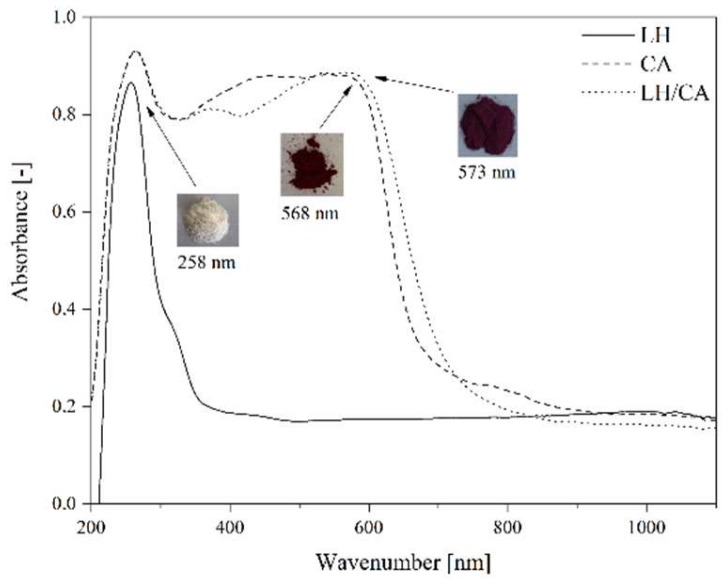
UV-Vis spectra of LH, carminic acid, and LH/CA (20%).

**Figure 7:**
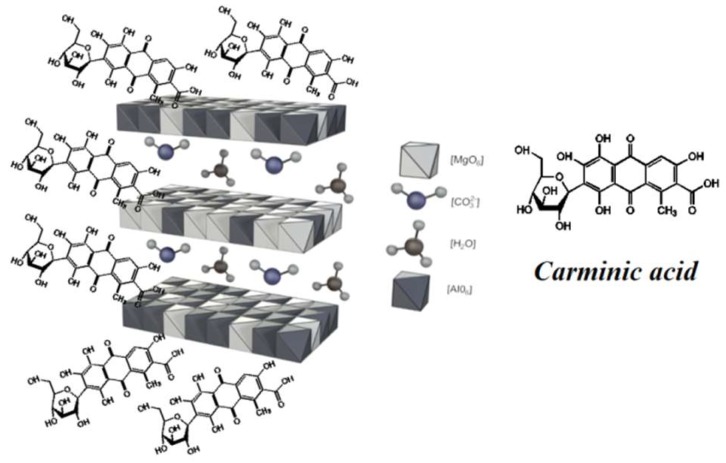
Proposed arrangement of carminic acid molecules in LH pigments.

Figure 6 shows the UV-Vis spectra of LH, CA, and LH/CA (20%). The absorption spectrum of CA showed a maximum at 568 nm. After the stabilization of CA on the LH matrix, the band shifted to 573 nm, most likely as a result of the dye-metal interaction. This spectral region is assigned to the absorption of CA, suggesting that CA did not decompose after interaction with the LH host. As a result, the modified LH/CA (20%) pigment showed a noticeable color change, from dark red to violet. Carminic acid is known to have three possible pKa values (2.8, 5.4, and 8.1). At pH 8, CA is completely deprotonated and violet tri-anionic molecules appear [37]. Because aluminum-magnesium hydroxycarbonate used in this study is basic in nature, the complexation most likely occurs between the carboxylic and hydroxy-keto groups in the CA structures and Mg ions present in the LH structure. It was concluded that the formation of organic-inorganic colorants seems similar to that for lake pigments, which are obtained by the precipitation of acid chromophores with salts of alkaline earth [15,32]. The possible arrangement of CA on an inorganic carrier is presented in Figure 7.

**Figure 8:**
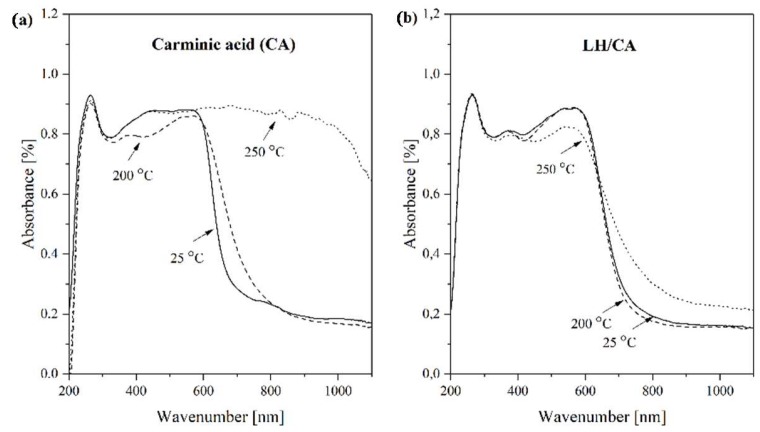
UV-Vis spectra of carminic acid and hybrid pigment exposed to different temperatures: (**a**) carminic acid and (**b**) LH/CA (20%).

**Figure 9:**
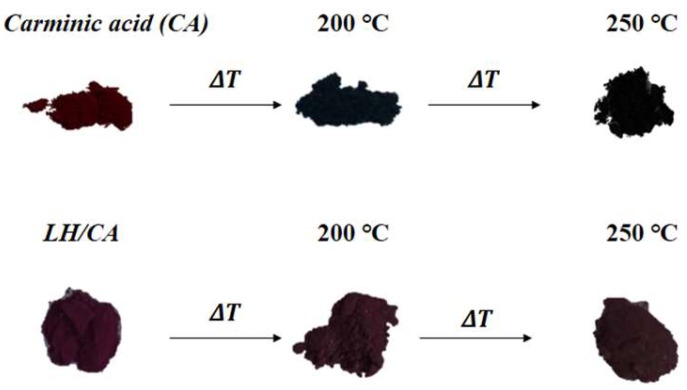
Color changes of carminic acid and LH/CA (20%).

The thermal stability of the LH-based pigments was assessed by heat treating the powders in an oven at 200 °C and 250 °C for 30 min. The UV-Vis spectra of the CA and hybrid pigments before and after thermal treatment are shown in Figure 8a,b. In general, all samples revealed similar spectra. Differences were observed during heat treatment, especially in the case of pure CA (Figure 8a). At 200 °C, the shape and *λ*_max_ of the CA spectra were changed, and the intensity of the absorption was lower, indicating the start of thermal degradation. These results are in line with TG data, as the main decomposition peak for pure chromophore was also detected at around 200 °C. Further heating at 250 °C caused a significant variation in the absorbance values, due to carbonization, and complete degradation of the organic dye structure (Figure 9). In contrast, the UV-Vis spectrum of the hybrid pigments shows no change at 200 °C, and decomposition of the chromophore was observed only when the sample was heated to 250 °C (Figure 8b). It can be concluded that the color stability of the LH pigments under elevated temperatures was a consequence of strong interactions between the Mg ions and the CA molecules, as was confirmed by XPS analysis.

**Figure 10:**
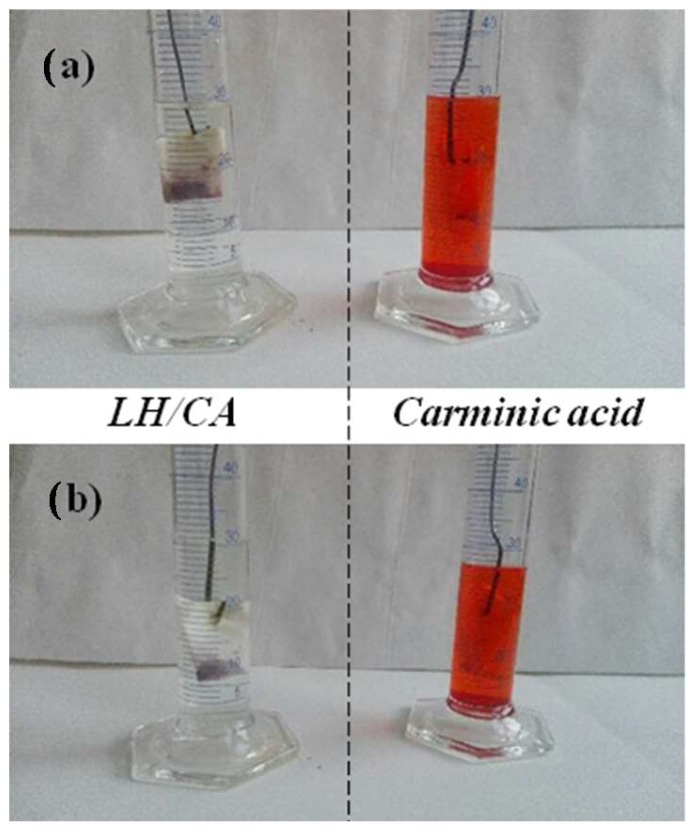
Digital images of carminic acid and LH/CA (20%) after 24 h of immersion in water (**a**) and acetone (**b**).

Figure 10 shows that the stabilization of CA on the LH host raised the solvent resistance of the obtained LH pigments considerably, while pure CA turned the solvent an intense red color after 24 h of immersion. This significant decrease in the solubility of CA can be attributed to strong interactions with the inorganic host, indicating successful transformation of the organic chromophore.

### 2.6. Scanning Electron Microscopy (SEM)

**Figure 11:**
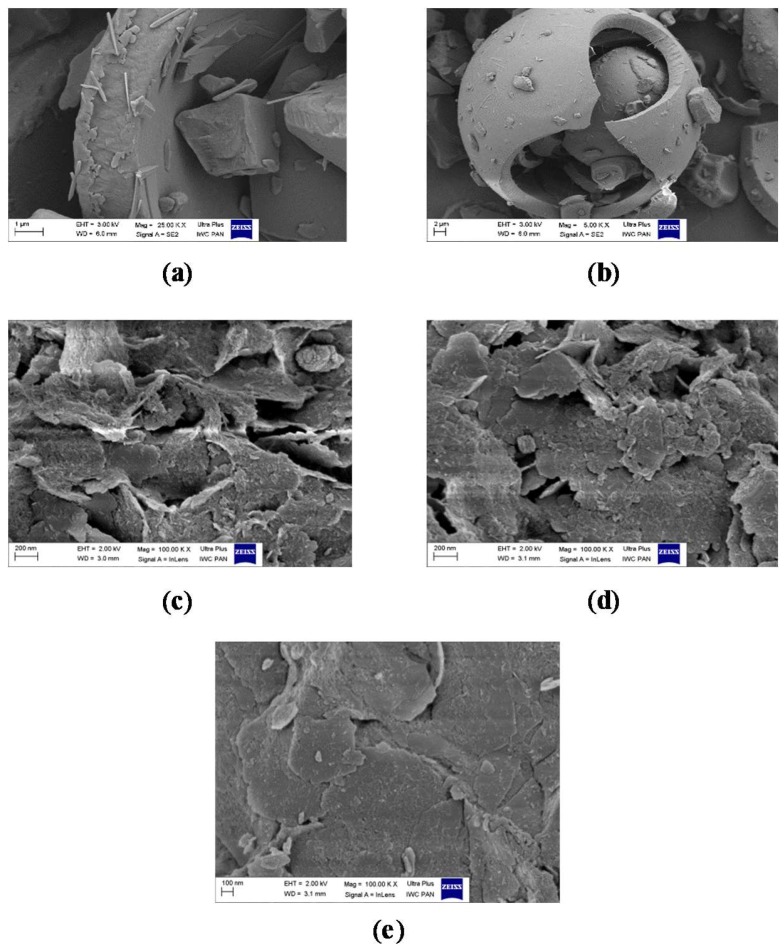
SEM morphology of: (**a**,**b**) carminic acid; (**c**) LH/CA (10%); (**d**) LH/CA (20%); (**e**) LH.

We also investigated the microscopic morphology of the hybrid pigments after modification with CA. The results are presented in Figure 11a–e. In contrast to the pure host, some roughness appeared on the LH surface after stabilization of the CA (Figure 11c,d). This is most likely associated with the deposition of carminic acid on the outer area of the LH structure. Unlike in our previous studies [36], the CA structures are not clearly distinguishable on the modified surface. This is due to the rather amorphic nature of the organic chromophore, which was also confirmed by XRD results.

### 2.7. Colorimetric and Structural Analysis of EN/Hybrid Pigment Compounds

**Figure 12:**
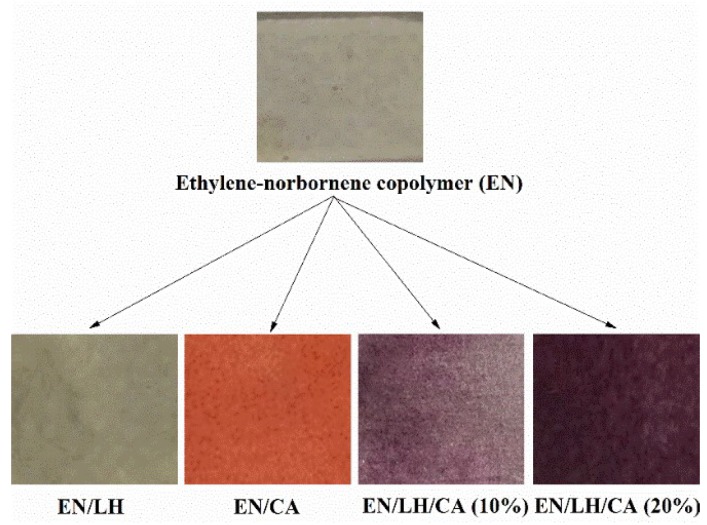
Digital photographs of ethylene-norbornene copolymer containing LH host, carminic acid, and hybrid pigments.

The color variation of the EN materials was studied in terms of L*, a*, and b* parameters (Table 3). A considerable difference in color can be seen between the reference EN samples (EN, EN/LH) and EN copolymer containing hybrid pigments and pure carminic acid (Figure 12). After the addition of hybrid colorants and CA dye, EN composites showed a significant decrease in the L* value, which is the parameter indicating lightness. In comparison to the EN copolymer colored with carminic acid, hybrid pigments provide an EN composite rather violet in shade. Therefore, the greatest changes for EN/hybrid pigment composites were observed for the a* parameter, corresponding to red-green colors in the CIE Lab system.

**Figure 13:**
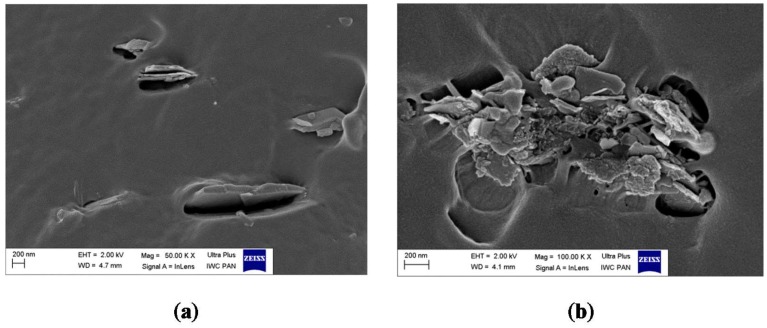
SEM micrographs of EN composites containing LH/CA (20%) (**a**,**b**).

To investigate the dispersion and the morphology of the hybrid pigment particles in the EN matrix, scanning electron microscopy (SEM) was performed. The images obtained are presented in Figure 13a,b. From the SEM microphotographs, it is evident that the LH/CA pigment retained its original plate-like shape, with side lengths as large as 400–700 nm after incorporation into the polymer matrix. Furthermore, the distribution and the compatibility of the hybrid pigment/EN matrix seem satisfactory, as no larger agglomerates were observed.

### 2.8. Flammability of EN/Hybrid Pigment Compounds

**Figure 14:**
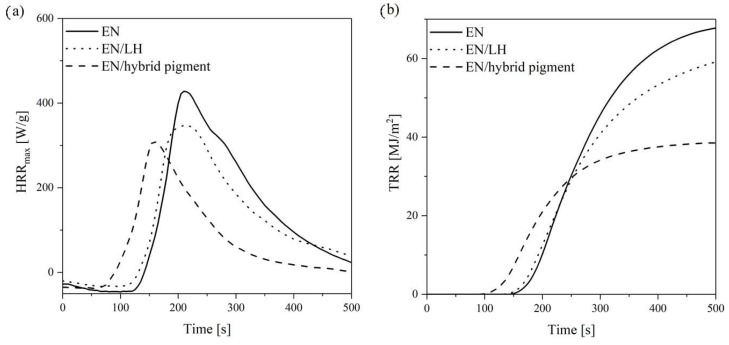
HRR (**a**) and THR (**b**) curves of EN, EN/LH, and EN/hybrid pigment copolymer.

Polymer composites filled with the hybrid pigment were prepared. The flame retardancy of the composites was evaluated using the cone calorimetry method. This technique approximates real fire conditions and is considered to be an ideal tool for assessing the flammability of polymer composites. The available flammability parameters of the cone calorimeter include the heat release rate (HRR), total heat release (THR), effective heat of combustion (EHC), and mass loss rate (MLR). The HRR and THR curves of the studied composites are given in Figure 14. The corresponding flammability data is presented in Table 4. It can be seen that the neat ethylene-norbornene copolymer exhibited very poor fire resistance, with a heat release peak of 427.8 kW/m^2^. However, the incorporation of 5 phr LH/CA pigment into the ethylene-norbornene composite reduced its flammability, as evidenced by the decrease in the HRR and THR parameters, by 55% and 44%, respectively, in comparison to the reference sample. Furthermore, the addition of hybrid pigment contributed to a slight reduction in the MLR parameter (from 6.9 to 6.5). It is known, that the use of an inorganic host (such as LH) is thought to promote the formation of an expanded carbonaceous char on the polymer, which prevents exposure to air. Therefore, the hybrid structure of the CA lakes may increase flame retardancy when applied in polymer composites. The increase in flame resistance for the EN composite with a pure LH host was less pronounced in comparison with the EN/hybrid pigment, as evidenced by higher values of considered flammability parameters. The higher efficiency of the hybrid colorants in terms of improving flame retardance of the EN copolymer may be attributed to the presence of an organic chromophore in the LH structure and was already observed in our previous work [36].

## 3. Materials and Methods 

### 3.1. Raw Materials

**Figure 15:**
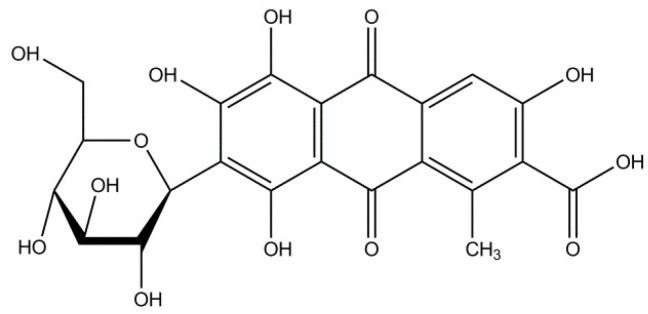
Chemical formula of carminic acid.

The LH carrier with an Al/Mg weight ratio of 70/30 was provided by Sasol GmbH (Hamburg, Germany). The chromophore—carminic acid (CA) (Figure 15), as well as the organic solvents toluene, cyclohexane, ethanol, and acetone, all of analytical grade, were purchased from Sigma-Aldrich (Schnelldorf, Germany). The ethylene-norbronene copolymer (EN) (40 wt % bound norbornene content) was supplied by TOPAS Advanced Polymers (Frankfurt-Höchst, Germany).

### 3.2. Hybrid Pigment Preparation

Carminic acid hybrid pigments with two CA concentrations (10% and 20%) were synthesized in aquatic medium. The LH was used as a hybrid pigment template. The LH/CA pigment (for 10% CA) was prepared as follows: 1 g of CA was dissolved in 200 mL of pure water with the addition of ethanol (50 mL). This solution was subjected to ultrasonication for 30 min. Next, 9 g of LA was added to the mixture and then the reaction system was stirred continuously at an elevated temperature (80 °C) for 3 h. The reaction product was filtered under a vacuum and then washed several times with deionized water until a colorless solution was observed. Finally, the hybrid pigment was dried in an oven at 70 °C for 24 h under a static air atmosphere (Binder, Tuttlingen, Germany).

### 3.3. Characterization of Powders

X-ray photoelectron spectroscopy (XPS) analysis was performed with the use of Mg *K_a_* (hν = 1253.6 eV) radiation. A Prevac (Rogów, Poland) electron spectrometer for chemical analysis equipped with a Scienta (Uppsala, Sweden) SES 2002 electron energy analyzer was used, operating at constant transmission energy (*E_p_* = 50 eV). The analysis chamber was evacuated to a pressure below 1 × 10^−9^ mbar. A powdered sample of the material was placed on a stainless steel sample holder. 

X-ray diffraction analysis (XRD) was performed using an Analytical Pert Pro MPD diffractometer (Malvern Panalytical Ltd., Royston, UK). The XRD patterns were collected in the Bragg-Brentano reflecting geometry with (Cu Kα) radiation, in the range of 2*θ* = 2–70°.

The thermal stability of the prepared hybrid pigments was evaluated using thermogravimetric analysis (TGA). Thermal analysis was conducted on a Thermogravimetric Analyzer TGA (TA Instruments, Greifensee, Switzerand) in a temperature range from 25 °C to 600 °C, with a heating rate of 10 °C/min. The measurements were processed in the presence of argon.

The solvent resistance of the LH/CA pigments was determined based on the PN-C-04406/1998 standard. The hybrid pigment powders were immersed in 20 mL of water and acetone.

Diffuse reflectance UV-Vis spectroscopy was performed using an Evolution 201/220 UV-Visible Spectrophotometer (Thermo Scientific, Waltham, MA, USA), with a spectral window from 1100 to 200 nm. 

The morphology of the pigments was investigated by scanning electron microscopy (SEM). The SEM micrographs were obtained using a LEO 1530 Gemini scanning electron microscope (Zeiss/LEO, Oberkochen, Germany). 

TOF-SIMS mass spectra were obtained using a TOF-SIMS IV secondary ion mass spectrometer (ION-TOF GmbH, Muenster, Germany). This apparatus is equipped with a high mass resolution time of flight analyzer and Bi_3_^+^ primary ion gun. Secondary ion mass spectra were recorded across an area of approximately 100 μm × 100 μm on the sample surface. The analyzed area was irradiated with pulses of 25 keV Bi_3_^+^ ions with a 10 kHz repetition rate and an average ion current of 0.4 pA. The measurement time was 30 s. Secondary ions emitted from the bombarded surface were mass separated and counted in a time of flight (TOF) analyzer. 

### 3.4. Preparation and Characterization of Polymer Composites

Ethylene-norbornene copolymer (EN) was used as a polymer matrix. The EN/hybrid pigment composites were prepared using a Brabender laboratory-scale measuring mixer N50 (Duisburg, Germany) at 110 °C. Each EN compound was processed with a rotor speed of 50 rpm for 10 min. After mixing, the polymer blends were pressed between two steel plates at 110 °C and under 15 MPa pressure for 10 min to obtain the final samples. The formulation of the prepared EN composites was as follows (phr—parts per hundred parts of rubber): 100 phr of ethylene-norbornene copolymer and 5 phr of hybrid pigment.

The flammability of the EN composites filled with LH/CA pigment was examined using a cone calorimeter from Fire Testing Technology Ltd. (East Grinstead, UK) according to the PN-ISO 5600 standard. Squared specimens (100 mm × 100 mm × 2 mm) were irradiated horizontally with a heat flux of 35 kW/m^2^.

The characterization of color performance of polymer composites was fixed in the spectrophotometric measurements using the CM-3600d spectrophotometer from Konica Minolta Sensing, Inc. (Osaka, Japan). The spectral range of this experiment was 360–740 nm. Color characteristics of the vulcanizates were defined by the colorimetric coordinates: brightness (L*), red-green component (a*), blue-yellow component (b*), and total change of color (∆E).

## 4. Conclusions

This paper has provided a detailed description of the production and properties of organic-inorganic pigments based on carminic acid (CA). Stabilization of CA on aluminum-magnesium hydroxycarbonate (LH) leads to pigments with an excellent resistance to acetone and water. This change may be explained by the formation of complexes between the dye molecules and magnesium ions. The formation of such complexes was confirmed by XPS studies, which showed that the position of the Mg 2p line maximum for the LH/CA composite shifted to a higher binding energy compared to that of pure LH. In respect to pure CA, the absorption band of the hybrid pigments in the visible region shifted towards higher wavelengths, producing a violet color and confirming metal-dye interaction. Moreover, the hybrid pigment obtained had an enhanced color stability under thermal treatment, as shown by UV-Vis measurements. The LH/CA pigment offers a promising solution for producing colored polymer composites with improved flame retardancy.

## Figures and Tables

**Table 1:** Quantitative evaluation of the surface composition of LA and LH-based pigments calculated based on XPS analysis.

Sample	Carbon	Oxygen	Magnesium	Aluminum
	At%			
LH	6	34	8	52
CA	68	32	-	-
LH/CA	12	25	9	54

**Table 2:** Thermogravimetric analysis of hybrid pigments.

Sample	Thermal Stability		
	T_5%_^1^ (°C)	T_10%_^1^ (°C)	T_20%_^1^ (°C)
CA	107	203	260
LH	119	205	329
LH/CA (10%)	105	205	334
LH/CA (20%)	105	206	335

^1^ Degradation temperatures of 5, 10, and 20% of sample.

**Table 3:** Color coordinates of the EN composites containing LH, carminic acid, and hybrid pigments.

Sample	Colorimetric Parameters ^1^			
	L*	a*	b*	∆E
EN	85.19	0.83	4.78	-
EN/LH	77.33	−1.11	5.99	8.19
EN/CA	56.77	34.58	20.58	46.87
EN/LH/CA (10%)	42.78	25.51	−2.42	49.60
EN/LH/CA (20%)	37.09	22.67	−2.27	53.30

^1^ L*—Lightness, a*—negative values for green and positive values for red, b*—negative values for and positive values for yellow, ΔE—total color change.

**Table 4:** Flammability results recorded using a cone calorimeter.

Sample	Flammability Parameters				
	HRR ^1^ (kW/m^2^)	HRR_MAX_ (kW/m^2^)	THR ^2^ (MJ/kg)	EHC ^3^ (MJ/kg)	MLR ^4^ (g/m^2^∙s)
EN	166.7	427.8	68.1	33.6	6.9
EN/LH	89.9	347.4	61.1	32.9	6.6
EN/hybrid pigment	75.3	308.7	38.5	23.5	6.5

^1^ Heat release rate; ^2^ Total heat release; ^3^ Effective heat of combustion; ^4^ Mass loss rate.
